# Effect of acute stress on working memory in pilots: Investigating the modulatory role of memory load

**DOI:** 10.1371/journal.pone.0288221

**Published:** 2024-01-25

**Authors:** Yaowei Liang, Xing Peng, Yu Meng, Yueying Liu, Qi Zhu, Zhi Xu, Jiazhong Yang

**Affiliations:** Institute of Aviation Human Factors and Cognitive Neuroscience, College of Flight Technology, Civil Aviation Flight University of China, Guanghan, Sichuan, China; University of Zurich, SWITZERLAND

## Abstract

Many practitioners, such as pilots, frequently face working memory (WM) demands under acute stress environments, while the effect of acute stress on WM has not been conclusively studied because it is moderated by a variety of factors. The current study investigated how acute stress affects pilots’ WM under different memory load conditions. There are 42 pilots conducting the experiments, consisting of 21 stress group participants experiencing the Trier Social Stress Test (TSST) and 21 control group participants experiencing the controlled TSST (C-TSST). Subsequently, both groups performed N-back tasks under three memory load conditions (0-back, 1-back, and 2-back). State Anxiety Inventory (S-AI), heart rate (HR), and salivary cortisol concentrations (SCC) were collected to analyze acute stress induction. The results revealed that (1) the TSST could effectively induce acute stress with higher S-AI, HR, and SCC; (2) higher memory load reduces WM accuracy (ACC) and delays response times (RT); (3) acute stress increases WM ACC under moderate load conditions (1-back task). These results suggest that acute stress may not necessarily impair WM and even improve WM performance under certain memory load conditions. Potential mechanisms of acute stress effects on WM and alternative explanations for the modulatory role of memory load consistent with the emotion and motivation regulation theory are discussed. These findings not only provide insight into the field of acute stress and WM but are also beneficial for pilot training and the development of stress management strategies.

## Introduction

Working memory (WM) is a cognitive system that is crucial for the temporary storage and manipulation of information [1], which plays an essential role in complex operations. In the highly demanding field of aviation, pilots often face unavoidable and acute stress due to weather conditions, technical malfunctions, and air traffic congestion. Acute stress caused by these factors may impair cognitive functions such as the WM [2, 3] through brief psychophysiological and behavioral changes. Therefore, understanding the relationship between acute stress and WM is critical to flight safety and performance.

The Trier Social Stress Test (TSST) is a well-established acute stress induction paradigm [4]. It consists of a videotaped free speech followed by a mental arithmetic task in front of a nonresponsive audience. It is an effective method to raise physiological and psychological responses to acute stress. In the physiology field, stress is primarily associated with the rapid response pathway of the sympathetic nervous system (SNS), which manifests as an increased heart rate. Slow response has been implicated in the activation of the hypothalamic-pituitary-adrenal (HPA) axis [5, 6] in terms of increased cortisol secretion.

The N-back task is a classic paradigm for measuring the WM [7]. Participants are asked to remember the stimulus presented N items before the current stimulus, where the value of “N” manipulates the level of memory load. In the aviation domain, WM plays a fundamental role in processing and storing information relevant to ongoing tasks, making it essential for pilots to respond effectively to instructions from air traffic controllers (ATC). The ability to execute these instructions correctly is also known as air-ground communication, which has been shown to be impaired by WM. For example, the Charkhi Dadri mid-air collision in Delhi in November 1996 was due to the pilots’ impaired WM, which caused the pilots to forget to stop their descent when they reached the target altitude assigned by ATC under acute stress conditions [8]. However, WM can also be impaired under very low acute stress conditions where the pilots fail to follow ATC instructions to enter the correct runway and step into disaster (Comair Flight 5191, 2006) [9].

To date, research on the effects of acute stress on WM has yielded mixed findings [10–12]. While the consensus is that acute stress can impair WM [13, 14], some studies suggest that certain levels of stress may improve WM performance [15, 16]. Further complicating this issue is the potential counteraction of memory load, which has been suggested to account for these discrepancies [17]. Thus, the specific effects of acute stress on pilots’ WM and the role of memory load in this relationship remain underexplored.

To address these gaps, the current research aims to investigate the specific effects of acute stress on pilots’ WM and to examine the potential moderating role of memory load. We used a modified version of the TSST to induce acute stress in pilots and an N-back task to measure their WM performance under different memory load conditions. In addition, the current study included a control group of pilots who were not exposed to acute stress in order to better understand the interaction between acute stress and memory load on WM.

We hypothesized that stress group participants should show a WM impairment compared to the control group; WM performance should decrease as memory load increases, regardless of acute stress induction. Furthermore, participants in the stress group should have a greater decline in WM performance in the higher memory load condition compared to the control group.

In summary, this study aims to further explore the complex relationship between acute stress and WM in pilots, particularly considering the potential moderating role of memory load. Given the critical importance of WM in aviation tasks and the potential impairment caused by acute stress, the results of this research may have practical implications for improving pilot training, stress management, and overall performance during flight.

## Materials and methods

### Participants

According to G*power calculations [18], a total sample size of at least 28 participants was required to provide sufficient power to detect a medium-sized effect (f = 0.25) with 80% power and a significance level of α = 0.05. We recruited forty-two healthy male pilots (aged 21–25 years) with normal or corrected-to-normal vision to participate in this study. Since the target population of the current study is pilots, a specific occupational group that often faces high-intensity work stress and challenges, we selected male participants because males are currently the majority in this profession. All participants received commercial aviation licenses from the Civil Aviation Administration of China (CAAC) and had an average of more than 230 flight hours in simulators and real aircraft. Participants were randomly assigned to stress and control groups prior to the experiment, and both groups were matched for flight hours, age, and education, with 21 participants in each group. They were paid a fee after the experiment. The Ethics Committee of the Civil Aviation Flight University of China approved this study. Each participant gave written informed consent. The authors did not have access to any information that could identify individual participants after data collection. The participants participated in the experiment in May 2022.

### Experimental design

#### Experimental procedure

**Fig 1:**
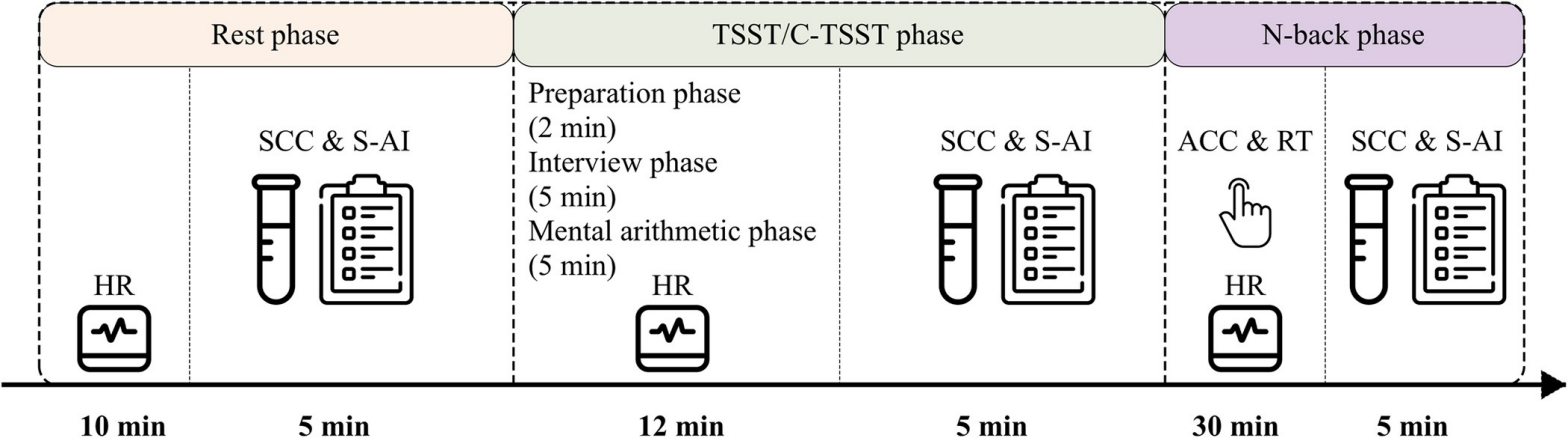
The procedure of the experiment. Participants experienced the Rest, TSST/C-TSST, and N-back phases, respectively. Salivary cortisol concentrations (SCC) and State Anxiety Inventory (S-AI) scores were collected at the end of each phase. Heart rate (HR) was excluded during the collection of SCC & S-AI scores. Accuracy (ACC) and reaction times (RT) were recorded during the N-back phase.

The experiment consisted of three phases: the Rest phase, the Trier Social Stress Test or controlled Trier Social Stress Test (TSST/C-TSST) phase, and the N-back phase (Fig 1).

*Rest phase*. In the first phase of the experiment, participants arrived at the laboratory and wore the portable devices. They sat undisturbed in a separate room for 10 minutes while their heart rate (HR) was recorded. They then completed basic demographic and state anxiety questionnaires and collected salivary cortisol. This was the first collection of salivary cortisol concentrations (SCC) and State Anxiety Inventory (S-AI) scores during the experiment and took approximately 5 minutes.

*TSST/C-TSST phase*. Participants in the stress group experienced a modified version of the TSST for 12 minutes. This included a 2-minute of preparation, a 5-minute interview, and a 5-minute mental arithmetic task. In contrast, participants in the control group experienced a similar procedure but without the acute stress elements, referred to as C-TSST. The HR was recorded during the TSST/C-TSST procedure. After that, there was the second collection of SCC and S-AI scores, and it took approximately 5 minutes. See the section on Acute stress induction for detailed procedure descriptions.

*N-back phase*. Participants should complete the N-back task for 30 minutes, which is designed to measure their WM. This task consists of three memory load conditions (0-back, 1-back, and 2-back). Each condition would be randomly presented to participants and take approximately 10 minutes. The accuracy (ACC), reaction times (RT), and HR were recorded during this task. After that, there was the third collection of SCC and S-AI scores, which took approximately 5 minutes.

In summary, SCC and S-AI scores were collected at the end of three different phases: the Rest phase, the TSST/C-TSST phase, and the N-back phase. While the HR was continuously recorded throughout the experiment using portable devices, the 5-minute intervals for collection of SCC and S-AI scores were excluded from the HR data analysis. The S-AI scores, HR, and SCC were selected as indicators of acute stress; ACC and RT were used as indicators of WM.

The study followed a mixed experimental design with two groups (stress and control) and three memory load conditions (0-back, 1-back, and 2-back), with “group” as a between-subjects variable and “memory load” as a within-subjects variable.

#### Acute stress induction

The traditional TSST procedures typically involve a short preparation time, a videotaped free speech, and a mental arithmetic task. To ensure the effectiveness of the acute stress induction for pilot participants, we adapted a modified version of the TSST to better match their professional characteristics and experiences. Below are detailed descriptions of the current TSST design.

In the preparation phase (2 min), the stress group participants could review the interview questions. The questions were selected from the International Civil Aviation Organization (ICAO) proficiency interview and the pilot qualification examination, which are used to form six questions. For example, Q: What are the symptoms of pilot hyperventilation? Q: Do you think it is necessary to carry out a cross-check? Why? In the interview phase (5 min), stress group participants were required to answer the questions randomly selected in front of two experts. They were informed beforehand that their answers would be videotaped and scored. In the mental arithmetic phase (5 min), stress group participants were asked to report the results quickly and accurately (e.g., starting from 2031, perform the mental arithmetic task in decreasing order of 18).

Throughout the TSST, stress group participants were informed that they would be videotaped and scored. Two experts (a psychologist and a flight instructor) wearing uniforms and work permits, recorded and scored the stress group participants’ performance and demeanor. Their serious facial expressions and occasional specific reactions, such as doubt and frowning, were designed to simulate a real-world evaluation scenario for pilots, thereby enhancing acute stress induction.

In the C-TSST, the control group participants conducted the procedure which was similar in format and duration to the TSST without its acute stress elements. The interview atmosphere was intentionally relaxed, with no formal attire, no videotaping, and no scoring. In contrast to the stress group participants who completed the TSST, the control group participants completed the control tasks without acute stress induction.

In the preparation phase (2 min), control group participants read material related to civil aviation. In the interview phase (5 min), they continued to read the material at a regular pace. In the mental arithmetic phase (5 min), they performed simple calculations, such as adding increments of 5 starting from 3. Control group participants were not videotaped and valued in C-TSST.

All participants were instructed to avoid strenuous exercise and alcohol consumption for 24 hours prior to the experiment and refrain from eating and smoking for 2 hours prior to the experiment.

#### Working memory measurement

**Fig 2:**
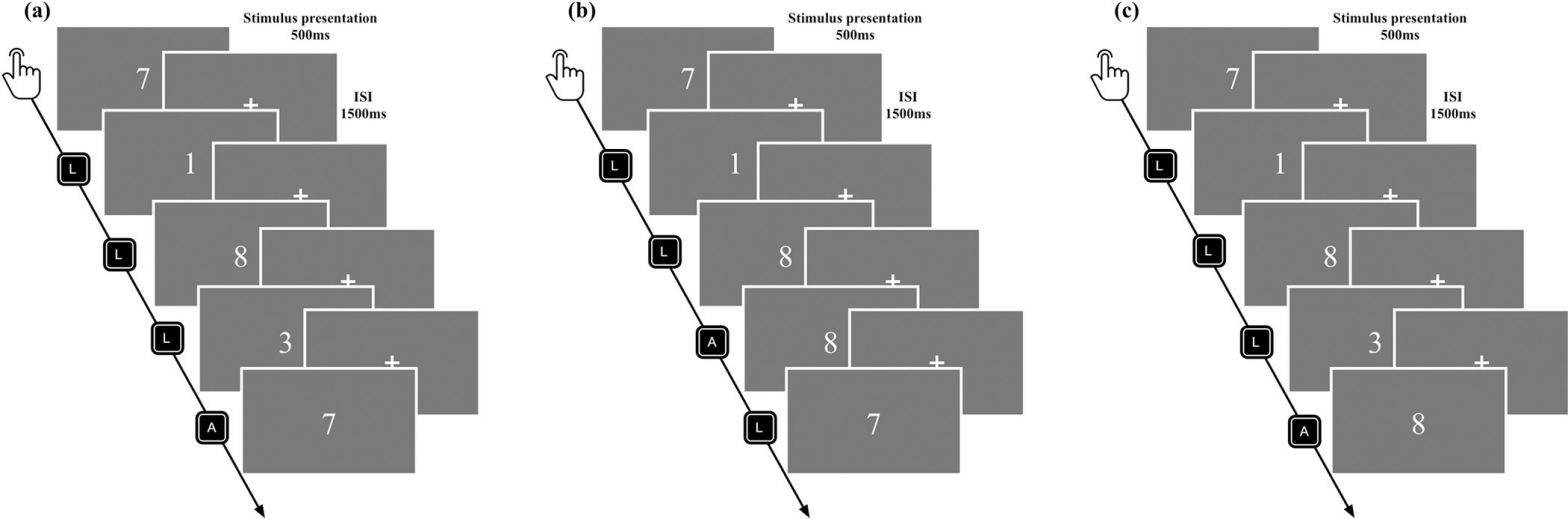
Example N-back tasks: (a) 0-back task, judge if the stimulus was the same as the first stimulus. (b) 1-back task, judge if the stimulus was the same as the previous stimulus. (c) 2-back task, judge if the stimulus was the same as the previous second stimulus.

Participants’ WM was measured with an N-back task (Fig 2). They performed the task in a quiet, dimly lit room at a distance of approximately 60 cm from the display. The stimuli were presented on a 17-inch display with a resolution of 1024*768 pixels and a refresh rate of 60 Hz using the software E-prime 2.0. The experimental stimuli were all presented on a black background, and the stimulus was a white digit (e.g., 1~9). The practice session consisted of 3 blocks of 60 stimulus trials with feedback. It was ensured that participants understood the instructions prior to the formal experiment. The formal experiment consisted of 3 blocks corresponding to low (0-back), moderate (1-back), and high (2-back) memory load, with each block having a total of 180 trials. Of these, 1/3 of the trials were digits that required participants to press the “A” key, and 2/3 required them to press the “L” key. Each stimulus was presented for 500 ms, followed by a “+” fixation for 1500 ms. Thus, participants should respond in 2000 ms. Participants should press the “A” and “L” keys with the index finger of their left and right hands.

Specifically, in the 0-back task, participants were asked to judge whether the current stimulus was the same as the first stimulus at the beginning of the trial, and if it was, to press the “A” key; if it was not, press the “L” key. In the 1-back task, participants were asked to judge whether the current stimulus appeared one position back in the sequence, and if so, to press the “A” key; if not, to press the “L” key. In the 2-back task, participants were asked to judge whether the current stimulus was the same as the second stimulus back in time, and if so, to press the “A” key; if not, to press the “L” key.

### Data analysis

A 2 (group: stress and control) × 3 (phase: Rest, TSST/C-TSST, N-back task) repeated measures ANOVA was performed on the data. To calculate the mean accuracy and reaction time for all participants, a 2 (group: stress and control) × 3 (memory load: 0-back, 1-back, 2-back) repeated measures ANOVA was performed on the data. Greenhouse-Geisser corrected p-values were used where appropriate.

## Results

### State Anxiety Inventory (S-AI) scores

**Fig 3:**
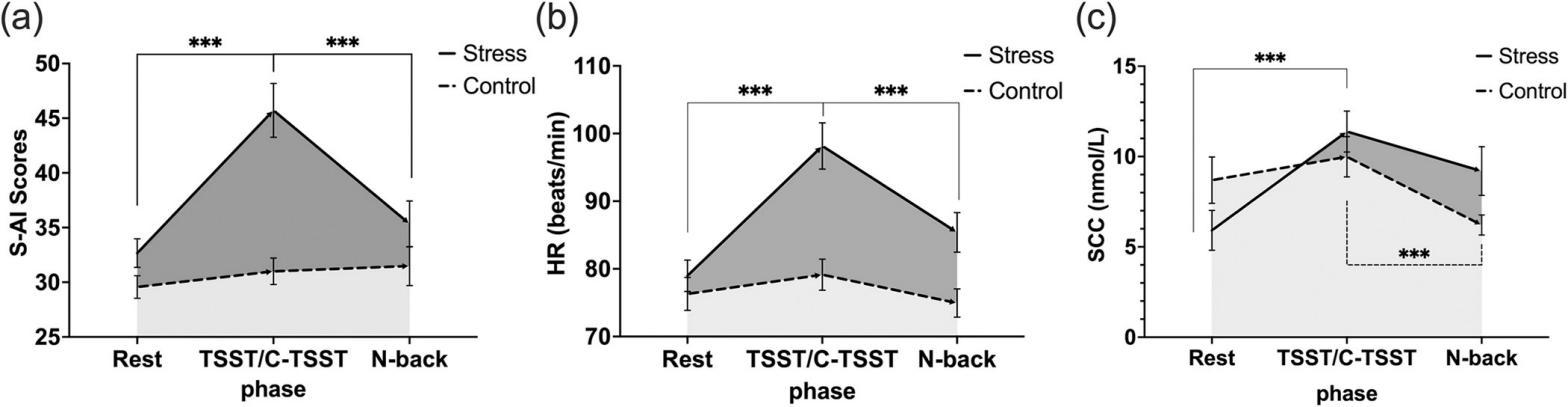
(a) S-AI scores, (b) heart rate (HR) (beats/min), and (c) salivary cortisol concentrations (SCC) (nmol/L) for stress and control groups at three phases, error bars show ± *SE*, ***p < 0.001, **p < 0.01, *p < 0.05.

The change in S-AI scores in three phases is shown in Fig 3(A). There was a significant main effect of group (*F*(1, 40) = 12.35, *p* < 0.001, *η*_*p*_^2^ = 0.24), and S-AI scores were significantly higher in the stress group than in the control group. The main effect of phase was also significant (*F*(2, 80) = 20.76, *p* < 0.001, *η*_*p*_^2^ = 0.34). The interaction between the group and phase was significant (*F*(2, 80) = 16.02, *p <* 0.001, *η*_*p*_^2^ = 0.29).

Simple effect analysis showed that there were no significant differences in S-AI scores between the three phases for the control group: Rest phase (*M* = 29.57, *SD* = 4.72), C-TSST phase (*M* = 31.00, *SD* = 5.57) and N-back phase (*M* = 31.48, *SD* = 8.18), *ps* > 0.05. In contrast, in the stress group, S-AI scores were significantly elevated in the TSST phase (*M* = 45.71, *SD* = 11.31) compared to the Rest phase (*M* = 32.68, *SD* = 5.96), *p* < .001. Moreover, S-AI scores in the TSST phase were also significantly higher than in the N-back phase (*M* = 35.33, *SD* = 9.65), *p* < 0.001. There was no significant difference in S-AI scores between the Rest phase and N-back phase, *p* = 0.931.

### Heart rate (HR)

The change in HR in three phases is shown in Fig 3(B). There was a significant main effect of group (*F*(1, 40) = 9.08 *p* = 0.004, *η*_*p*_^2^ = 0.19). HR was significantly higher in the stress group than in the control group. The main effect of phase was also significant (*F* (2, 80) *=* 78.20, *p* < 0.001, *η*_*p*_^2^ = 0.66). Participants’ HR was significantly higher in the TSST/C-TSST phase compared to both the Rest phase and N-back phase. The interaction between the group and phase was also significant (*F*(2, 80) = 39.25, *p* < 0.001, *η*_*p*_^2^ = 0.5).

Simple effect analysis showed that for the control group, no significant differences in HR were found between the Rest phase (*M* = 76.29, *SD* = 11.23), the C-TSST phase (*M* = 79.14, *SD* = 10.52), and the N-back phase (*M* = 74.96, *SD* = 9.57), *ps* > 0.05. In contrast, HR in the stress group showed a significant difference between the Rest phase (*M* = 78.97, *SD* = 10.65) and the TSST phase (*M* = 98.16, *SD* = 15.67), *p* < 0.001. Additionally, there was a significant difference between the TSST phase and the N-back phase (*M* = 85.4, *SD* = 13.45), *p* < 0.001, and a significant difference between the Rest phase and N-back phase, *p* < 0.001.

### Salivary cortisol concentrations (SCC)

The change in SCC in three phases is shown in Fig 3(C). There was a significant main effect of group (*F*(1, 40) = 151.53, *p* < 0.001, *η*_*p*_^2^ = 0.79). The SCC was significantly higher in the stress group than in the control group. The main effect of phase was significant (*F*(2, 80) *=* 15.89 *p* < 0.001, *η*_*p*_^2^ = 0.29). The SCC was significantly higher in the TSST/C-TSST phase compared to both the Rest phase and N-back phase. The interaction between group and phase was also significant (*F*(2, 80) = 10.29, *p* < 0.001, *η*_*p*_^2^ = 0.2).

Simple effect analysis showed that for the control group, while no significant differences in SCC were found between the Rest phase (*M* = 8.69, *SD* = 5.88) and the C-TSST phase (*M* = 9.98, *SD* = 5.10), *p* > 0.05, a significant difference was found between the C-TSST phase and the N-back phase (*M* = 6.21, *SD* = 2.54), *p* < 0.001. In contrast, for the stress group, there was a significant difference between the Rest phase (*M* = 5.92, *SD* = 5.06) and the TSST phase (*M* = 11.38, *SD* = 5.19), *p* < 0.001, and a significant difference between the Rest phase and N-back phase (*M* = 9.20, *SD* = 6.2), *p* < 0.001.

### Behavioral data

#### Accuracy (ACC)

**Fig 4:**
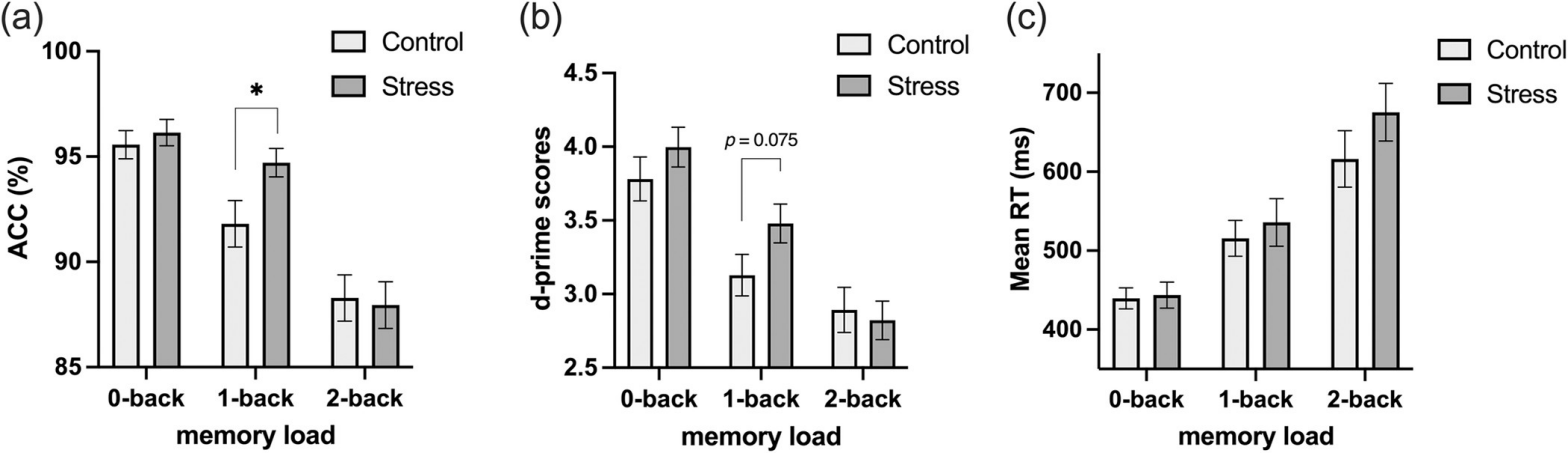
(a) accuracy (ACC), (b) d-prime scores (d’), and (c) Mean reaction times (RT) in the 0-back, 1-back, and 2-back conditions, error bars show ± *SE*, ***p < 0.001, **p < 0.01, *p < 0.05.

The main effect of memory load was significant (*F*(2, 80) = 69.94, *p* < 0.001, *η*_*p*_^2^ = 0.64). Accuracy in the 0-back condition (*M* = 0.96, *SD* = 0.03) was significantly higher than in the 1-back condition (*M* = 0.93, *SD* = 0.04), *p* < 0.001, and 2-back condition (*M* = 0.88, *SD* = 0.05), *p* < 0.001, and it was significantly higher in 1-back tasks than in 2-back tasks, *p* < 0.001. The main effect of the group was not significant (*F*(1, 40) = 1.04, *p* = 0.31). The interaction of group and memory load was significant (*F*(2, 80) = 3.15, *p* = 0.048, *η*_*p*_^2^ = 0.07). Further analysis revealed that the stress group had significantly higher accuracy (*M* = 0.95, *SD* = 0.03) than the control group (*M* = 0.92, *SD* = 0.05) in the 1-back task, *p* = 0.03, see Fig 4(A).

The d-prime score (d’), a measure of discrimination accuracy, was calculated to further validate the accuracy results. The main effect of memory load was significant (*F*(2, 80) = 58.14, *p* < 0.001, *η*_*p*_^2^ = 0.592). The d’ in the 0-back condition (*M* = 3.89, *SD* = 0.56) was significantly higher than in the 1-back condition (*M* = 3.30, *SD* = 0.64), *p* < 0.001, and 2-back condition (*M* = 2.86, *SD* = 0.65), *p* < 0.001, and it was significantly higher in 1-back tasks than in 2-back tasks, *p* < 0.001. The main effect of the group was not significant (*F*(1, 40) = 1.034, *p* = 0.32). The interaction of group and memory load was marginally significant (*F*(2, 80) = 2.51, *p* = 0.088, *η*_*p*_^2^ = 0.06). Further analysis revealed that the stress group had a slightly higher d’ (*M* = 3.48, *SD* = 0.61) than the control group (*M* = 3.12, *SD* = 0.65) in the 1-back task, *p* = 0.075, but not significantly in the 0-back and 2-back conditions, see Fig 4(B). While the d’ results did not reach the same level of significance as the accuracy results, they did reflect the trend that the stress group’s discrimination ability slightly surpassed that of the control group in the 1-back condition.

#### Reaction time (RT)

There was a significant main effect of memory load (*F*(2, 80) = 75.49, *p* < 0.001, *η*_*p*_^2^ = 0.6). The RT in the 1-back condition (*M* = 525.67, *SD* = 121.50) was significantly longer than that in the 0-back condition (*M* = 441.64, *SD* = 68.14), *p* < 0.001, and the RT in the 2-back condition (*M* = 645.86, *SD* = 166.36) was significantly longer than that in the 1-back condition, *p* < 0.001, and in 0-back condition, *p* < 0.001. The main effect of the group was not significant (*F(*1, 40) = 0.69, *p* = 0.41). The interaction of group and memory load was not significant (*F*(2, 80) = 1.43, *p* = 0.26), see Fig 4(C).

## Discussion

The present study focused on the effect of acute stress on pilots’ WM with the modulatory role of memory load. The acute stress was successfully induced by the modified version of the Trier Social Stress Test (TSST), as evidenced by increased self-reported anxiety, an accelerated heart rate, and elevated salivary cortisol concentrations in the stress group. In addition, as we hypothesized, increasing memory load was associated with slower reaction times and decreasing accuracy. However, in higher load conditions, the control group did not necessarily perform better than the stress group and performed even less accurately in the moderate memory load condition.

### The effect of acute stress on working memory

In the present study, we used three different indicators to characterize acute stress in individuals. The results showed that these psychological, electrophysiological, and bio-physiological indicators all similarly described individuals’ levels of TSST-induced acute stress, providing additional evidence to the field of acute stress and the relationship between different measurements.

Although the mechanism of the effect of acute stress on WM is not fully conclusive, especially considering that acute stress can be induced by different methods [4, 19, 20], most of the existing research suggests that this effect is related to the activity of the prefrontal cortex (PFC). For instance, Amy F. T. Arnsten and Patricia S. Goldman-Rakic have investigated the effects of dopamine D1 receptors on cognitive performance among non-human primates. Their research reveals a correlation between PFC activity and cognitive abilities [21, 22]. It is suggested that excessive dopamine release during stress may impair PFC function, a dynamic described by an inverted-U shape, depicting that either an excess or deficiency in PFC activity—such as that caused by varying levels of dopamine D1 receptor stimulation—can be detrimental to cognitive performance [23, 24]. They concluded that there is a narrowly defined optimal range of physiological conditions, situated within the everyday spectrum of experiences like fatigue and moderate stress, under which cognitive functions are best performed in humans [25].

Arnsten & Goldman-Rakic’s suggestions were later supported by other research. For example, Itthipuripat et al. (2013) suggested a correlation between PFC activity and the successful performance of a WM task [26]. They found that during tasks that required participants to manipulate the information in their WM, there was a significant increase in frontal theta activity compared to tasks that did not require manipulation, and it was even more pronounced in individuals who performed better on the tasks.

In addition, Gärtner’s study further found that acute stress attenuates prefrontal theta activity associated with WM tasks [27]. Notably, while all of the above studies used different methods of acute stress induction (e.g., high-intensity sound in Arnsten & Goldman-Rakic’s research, negative movie clips in Gärtner’s research), these studies suggested that acute stress could directly affect PFC activity involved in cognitive tasks, resulting in either hyperactivity or inhibition of activity, potentially impairing performance on WM tasks.

However, there is conflicting evidence regarding the quantitative analysis of the effects of acute stress on WM. In contrast to the neurological evidence, acute stress at the behavioral level does not necessarily lead to reduced WM performance [17, 28, 29]. A discussion of this point relates to the second finding of the present study.

### The modulatory role of memory load

The second key finding of this study is that the effects of acute stress on WM are moderated by other factors, such as memory load. In the present experiment, WM was not significantly impaired by acute stress as observed at the behavioral level. On the contrary, participants in the stress group showed better WM performance under moderate memory load conditions. Furthermore, this improvement in WM is less likely to be biased in behavioral outcomes due to ceiling effects or increased attention, as there were no significant differences in reaction times between the control and stress groups in any of the three memory load conditions.

This finding has some commonalities with some previous research, where the effect of acute stress on WM was modulated by several factors [15, 27]. For example, Cornelisse et al. (2011) found that the effects of acute stress on WM were influenced by gender factors [15]. Their study suggests that the same acute stress induction method will cause a different change in salivary cortisol and alpha-amylase levels in males and females, leading to different WM outcomes. Even though the authors emphasize that the autonomic nervous system (ANS) and the HPA axis are activated by stress to release different cortisol concentrations in males and females, our findings suggest that cortisol concentration might not necessarily be related to WM differences because as salivary cortisol concentration increased the overall WM did not decrease, Fig 3(C). Similarly controversial was the research of Duncko et al. (2009), who found that the acute stress induced by cold pressure stress (CPS) caused a decrease in WM performance (lower accuracy and shorter reaction times), but without an increase in cortisol concentration [16]. Therefore, we suggested that the dependent variable could be directly affected by the moderating factor, which in this case is memory load.

It sounds reasonable that a higher memory load could impair WM performance because individuals have a limited capacity to store information. However, the effects of acute stress and memory load on WM performance may not be observed simultaneously. For example, studies have found that acute stress may only impair WM when memory load is extremely high, because a lower memory load task, compared to more complex paradigms, places relatively low demands on WM and may not be sensitive to small changes in performance caused by acute stress [10]. However, other studies suggest that the cognitive capacity demand on WM may actively reduce cortical processing of nociceptive stress input, as WM impairment was only seen in the lower memory load condition [17].

Although the two studies mentioned above have differences in the measurement of WM and the definition of memory load, memory load might somewhat modulate the effect of acute stress on WM, as our results provide evidence that acute stress can even improve WM under moderate load conditions. The alternative explanation could be the relative intensity of PFC activation.

As mentioned above, both hyperactivity and inhibition of PFC activation could impair the ability to perform cognitive tasks [22, 26, 27], a phenomenon depicted by the inverted-U shaped response observed in certain biological systems, which signifies that stress beyond our control has the potential to disrupt or possibly augment PFC functionality. Consequently, performance would be affected differently because different memory load tasks may be associated with different intensities of the PFC activation [30, 31]. If the intensity change caused by acute stress deviates from the cognitive capacity requirement of memory load, the WM performance could appear either impaired or enhanced. As in the current study, acute stress improves WM in a moderate load condition but not in other load conditions.

The current study with pilots provides similar evidence of the inverted-U shape between stress and WM performance. In part, this may be because stress levels and cognitive demands, such as memory load, simultaneously alter pilots’ physiological responses. Given their frequent exposure to stress, both from interpersonal dynamics in the cockpit and from the actual demands of the task, pilots may adapt to have a lower baseline SNS activation in stress-free situations and a broader optimal range of physiological conditions in stressful environments. Consequently, pilots may benefit from a certain level of stress induction, thereby exhibiting the characteristic inverted-U shaped curve in their cognitive performance.

In another respect, our recommendation is somewhat compatible with another theory of how emotion and motivation regulate executive control [32]. As emotional and motivational states (e.g., the anticipation of a reward) might increase activity in the dorsolateral prefrontal cortex (dlPFC), the upcoming memory load (e.g., the anticipation of a more challenging task) might also modulate PFC activity, leading to discrepancies in the effects of acute stress on WM under different memory load conditions. Therefore, in both cases, future research could pay attention to changes in PFC activity caused by acute stress or other factors when examining WM performance, which may be beneficial in addressing the discrepancies.

### Limitations and future directions

The first limitation of our studies is the dominance of male participants in our sample, which restricts the generalizability of our findings, as gender differences may influence the impact of acute stress on WM. However, as one of our goals is to provide practical implications for pilot training and stress management strategies, we select the males as participants, who are majority practitioners in the current aviation industry. Second, while the stress induction method was successful in inducing acute stress, it may not fully represent the variety and intensity of stress sources that pilots may face in real-world tasks because pilots may face regulatory and safety requirements in real-world flight. Third, our manipulation and measurement of WM provide only limited perspectives on complex cognitive function. Future research could explore additional cognitive tasks and measures, as well as the acute stress induction method.

## Conclusion

The current study provides insight into the relationship between acute stress and WM performance in pilots, highlighting that memory load may modulate the effect of acute stress on WM, as acute stress may improve WM in a moderate memory load condition. Our findings suggest that both acute stress and memory load may influence WM performance, and the interaction of these factors determines whether WM is impaired or improved.

## References

[1] Cowan N. The many faces of working memory and short-term storage. Psychonomic Bulletin & Review. 2017;24(4):1158–70. doi: 10.3758/s13423-016-1191-6 . 27896630

[2] Smeets T. Acute stress impairs memory retrieval independent of time of day. Psychoneuroendocrinology. 2011;36(4):495–501. doi: 10.1016/j.psyneuen.2010.08.001 . 20800361

[3] Shields GS, Sazma MA, Yonelinas AP. The effects of acute stress on core executive functions: A meta-analysis and comparison with cortisol. Neuroscience & Biobehavioral Reviews. 2016;68:651–68. doi: 10.1016/j.neubiorev.2016.06.038 . 27371161 PMC5003767

[4] Kirschbaum C, Pirke KM, Hellhammer DH. The ’Trier Social Stress Test’—a tool for investigating psychobiological stress responses in a laboratory setting. Neuropsychobiology. 1993;28(1–2):76–81. doi: 10.1159/000119004 . 8255414

[5] Kuhlmann S, Piel M, Wolf OT. Impaired memory retrieval after psychosocial stress in healthy young men. Journal of Neuroscience. 2005;25(11):2977–82. doi: 10.1523/JNEUROSCI.5139-04.2005 . 15772357 PMC6725125

[6] Dickerson SS, Kemeny ME. Acute stressors and cortisol responses: a theoretical integration and synthesis of laboratory research. Psychol Bull. 2004;130(3):355–91. doi: 10.1037/0033-2909.130.3.355 . 15122924

[7] Owen AM, Mcmillan KM, Laird AR, Bullmore EE. N-back working memory paradigm: a meta-analysis of normative functional neuroimaging studies. Human Brain Mapping. 2005;25(1):46–59. doi: 10.1002/hbm.20131 . 15846822 PMC6871745

[8] Lahoti RC. Report of Court of Inquiry on Mid-Air Collision Between Saudi Arabian Boeing 747 and Kazakhstan IL-76 on 12th November, 1996 Near Delhi–India (Charkhi-Dadri, Haryana). 1997.

[9] NTSB. Attempted takeoff from wrong runway, Comair Flight 5191, Bombardier CL‐600‐2B19, N431CA, Lexington, Kentucky, August 27, 2006. 2007.

[10] Schoofs D, Wolf OT, Smeets T. Cold pressor stress impairs performance on working memory tasks requiring executive functions in healthy young men. Behavioral Neuroscience. 2009;123(5):1066–75. doi: 10.1037/a0016980 . 19824773

[11] Woodcock EA, Greenwald MK, Khatib D, Diwadkar VA, Stanley JA. Pharmacological stress impairs working memory performance and attenuates dorsolateral prefrontal cortex glutamate modulation. Neuroimage. 2019;186:437–45. doi: 10.1016/j.neuroimage.2018.11.017 . 30458306 PMC6491044

[12] Jiang C, Rau PLP. Working memory performance impaired after exposure to acute social stress: The evidence comes from ERPs. Neuroscience Letters. 2017;658:137–41. doi: 10.1016/j.neulet.2017.08.054 . 28851617

[13] Lupien SJ, Maheu F, Tu M, Fiocco A, Schramek TE. The effects of stress and stress hormones on human cognition: Implications for the field of brain and cognition. Brain and cognition. 2007;65(3):209–37. doi: 10.1016/j.bandc.2007.02.007 . 17466428

[14] Schoofs D, Preuß D, Wolf OT. Psychosocial stress induces working memory impairments in an n-back paradigm. Psychoneuroendocrinology. 2008;33:643–53. doi: 10.1016/j.psyneuen.2008.02.004 . 18359168

[15] Cornelisse S, van Stegeren AH, Joëls M. Implications of psychosocial stress on memory formation in a typical male versus female student sample. Psychoneuroendocrinology. 2011;36(4):569–78. doi: 10.1016/j.psyneuen.2010.09.002 . 20933337

[16] Duncko R, Johnson L, Merikangas K, Grillon C. Working memory performance after acute exposure to the cold pressor stress in healthy volunteers. Neurobiol Learn Mem. 2009;91(4):377–81. doi: 10.1016/j.nlm.2009.01.006 . 19340949 PMC2696884

[17] Legrain V, Crombez G, Plaghki L, Mouraux A. Shielding cognition from nociception with working memory. Journal of Psychological Science. 2013;49(7):1922–34. doi: 10.1016/j.cortex.2012.08.014 . 23026759

[18] Faul F, Erdfelder E, Lang AG, Buchner A. G*Power 3: a flexible statistical power analysis program for the social, behavioral, and biomedical sciences. Behav Res Methods. 2007;39(2):175–91. doi: 10.3758/bf03193146 . 17695343

[19] Ishizuka K, Hillier A, Beversdorf DQ. Effect of the cold pressor test on memory and cognitive flexibility. Neurocase. 2007;13(3):154–7. doi: 10.1080/13554790701441403 . 17786773

[20] Yurtsever T, Schilling TM, Kölsch M, Turner JD, Meyer J, Schächinger H, et al. The acute and temporary modulation of PERIOD genes by hydrocortisone in healthy subjects. Chronobiol Int. 2016;33(9):1222–34. doi: 10.1080/07420528.2016.1211668 . 27485028

[21] Arnsten AF, Cai JX, Murphy BL, Goldman-Rakic PS. Dopamine D1 receptor mechanisms in the cognitive performance of young adult and aged monkeys. Psychopharmacology (Berl). 1994;116(2):143–51. doi: 10.1007/BF02245056 . 7862943

[22] Arnsten AF, Goldman-Rakic PS. Noise stress impairs prefrontal cortical cognitive function in monkeys: evidence for a hyperdopaminergic mechanism. Arch Gen Psychiatry. 1998;55(4):362–8. doi: 10.1001/archpsyc.55.4.362 . 9554432

[23] Vijayraghavan S, Wang M, Birnbaum SG, Williams GV, Arnsten AF. Inverted-U dopamine D1 receptor actions on prefrontal neurons engaged in working memory. Nat Neurosci. 2007;10(3):376–84. doi: 10.1038/nn1846 . 17277774

[24] Williams GV, Goldman-Rakic PS. Modulation of memory fields by dopamine D1 receptors in prefrontal cortex. Nature. 1995;376(6541):572–5. doi: 10.1038/376572a0 . 7637804

[25] Arnsten AF. The neurobiology of thought: the groundbreaking discoveries of Patricia Goldman-Rakic 1937–2003. Cereb Cortex. 2013;23(10):2269–81. doi: 10.1093/cercor/bht195 . 23926115 PMC3767966

[26] Itthipuripat S, Wessel JR, Aron AR. Frontal theta is a signature of successful working memory manipulation. Experimental Brain Research. 2013;224(2):255–62. doi: 10.1007/s00221-012-3305-3 . 23109082 PMC3536917

[27] Gärtner M, Rohde-Liebenau L, Grimm S, Bajbouj M. Working memory-related frontal theta activity is decreased under acute stress. Psychoneuroendocrinology. 2014;43:105–13. doi: 10.1016/j.psyneuen.2014.02.009 . 24703176

[28] Han L, Liu Y, Zhang D, Jin Y, Luo Y. Low-arousal speech noise improves performance in N-back task: an ERP study. PLoS One. 2013;8(10):e76261. doi: 10.1371/journal.pone.0076261 . 24204607 PMC3799905

[29] Zhang Y, Luo Y, Sun L, Zhao S, Li H. The Acute Stress Interference Effect on Working Memory Depends on Load: Electrophysiological Evidences. Journal of Psychological Science. 2015;38(1):42–7.

[30] Porcelli AJ, Cruz D, Wenberg K, Patterson MD, Biswal BB, Rypma B. The effects of acute stress on human prefrontal working memory systems. Physiol Behav. 2008;95(3):282–9. doi: 10.1016/j.physbeh.2008.04.027 . 18692209

[31] Qin S, Hermans EJ, van Marle HJ, Luo J, Fernández G. Acute psychological stress reduces working memory-related activity in the dorsolateral prefrontal cortex. Biol Psychiatry. 2009;66(1):25–32. doi: 10.1016/j.biopsych.2009.03.006 . 19403118

[32] Pessoa L. How do emotion and motivation direct executive control? Trends in Cognitive Sciences. 2009;13(4):160–6. doi: 10.1016/j.tics.2009.01.006 . 19285913 PMC2773442

